# Supplementary material for: Prevalence and predictors of childfree people in developing countries
Source: PLoS One. 2025 Nov 12;20(11):e0333906. doi: 10.1371/journal.pone.0333906 (PMC12611150; doi:10.1371/journal.pone.0333906)
Supplement: S1 Text — (PDF) [file pone.0333906.s001.pdf]

Supplementary Information for:
"Prevalence and predictors of childfree people in developing countries"

Zachary P. Neal and Jennifer Watling Neal

S1 Country characteristics and estimated childfree prevalence

		Number of		Sample Proportion							Country Variables			Childfree Prevalence	
**Country**	**Year**	**Respondents**	**Childfree**	**Women**	**Single**	**Urban**	**Under 22**	**Age 22-29**	**Age 30-39**	**Over 39**	**HDI**	**GII**	**GFS**	**Married Women**	**Single Women**
Afghanistan	2016	39592	82	0.732	0.000	0.234	0.113	0.332	0.318	0.236	0.479	0.692	0.240	0.003	—
Albania	2018	16924	333	0.638	0.303	0.455	0.192	0.202	0.233	0.372	0.796	0.145	0.680	0.002	0.025
Angola	2016	19994	169	0.719	0.370	0.625	0.320	0.291	0.224	0.165	0.591	0.548	0.280	0.001	0.027
Armenia	2016	8719	55	0.690	0.319	0.573	0.172	0.273	0.306	0.248	0.773	0.295	0.460	< 0.001	0.010
Bangladesh	2022	28999	36	1.000	0.000	0.352	0.124	0.279	0.350	0.247	0.670	0.498	0.390	0.001	—
Benin	2018	23182	153	0.677	0.281	0.443	0.275	0.275	0.249	0.202	0.506	0.658	0.820	0.002	0.022
Burkina Faso	2021	24775	59	0.701	0.274	0.357	0.284	0.237	0.264	0.215	0.445	0.627	0.540	0.001	0.003
Burundi	2017	24562	117	0.696	0.375	0.218	0.300	0.263	0.246	0.191	0.423	0.508	0.190	< 0.001	0.013
Cambodia	2022	28286	822	0.689	0.265	0.360	0.224	0.231	0.328	0.217	0.596	0.484	0.240	0.002	0.070
Cameroon	2019	21052	102	0.679	0.372	0.533	0.285	0.251	0.227	0.237	0.581	0.565	0.220	0.001	0.006
Chad	2015	21906	54	0.775	0.178	0.251	0.274	0.263	0.265	0.199	0.388	0.718	0.210	< 0.001	0.007
Colombia	2016	74327	1640	0.520	0.376	0.728	0.300	0.204	0.223	0.273	0.758	0.417	0.630	0.007	0.079
Congo Democratic Republic	2014	27105	163	0.686	0.270	0.358	0.295	0.263	0.245	0.197	0.450	0.641	0.200	0.001	0.021
Cote d'Ivoire	2021	22331	44	0.662	0.341	0.493	0.274	0.231	0.281	0.214	0.530	0.613	0.440	0.001	0.007
Egypt	2014	21657	11	1.000	0.000	0.442	0.074	0.316	0.350	0.260	0.688	0.541	0.310	< 0.001	—
Ethiopia	2016	27757	798	0.554	0.321	0.324	0.281	0.268	0.255	0.197	0.465	0.521	0.150	0.005	0.089
Gabon	2021	16140	102	0.583	0.398	0.700	0.246	0.232	0.247	0.275	0.704	0.532	0.220	0.001	0.015
Gambia	2020	15865	15	0.725	0.323	0.562	0.291	0.266	0.258	0.185	0.492	0.589	0.460	< 0.001	0.004
Ghana	2023	21988	55	0.682	0.359	0.481	0.257	0.250	0.270	0.222	0.602	0.512	0.800	0.001	0.005
Guatamala	2015	37017	445	0.699	0.340	0.433	0.301	0.250	0.242	0.208	0.629	0.525	0.550	0.001	0.026
Guinea	2018	14551	167	0.726	0.281	0.379	0.290	0.238	0.252	0.220	0.464	0.628	0.410	0.006	0.041
Haiti	2017	24143	121	0.595	0.429	0.377	0.300	0.241	0.225	0.234	0.557	0.626	0.390	< 0.001	0.009
India	2020	820342	24535	0.878	0.260	0.249	0.230	0.257	0.274	0.238	0.638	0.453	0.710	0.003	0.095
Indonesia	2017	58421	144	0.829	0.200	0.528	0.172	0.204	0.312	0.312	0.708	0.462	0.650	< 0.001	0.004
Jordan	2023	15328	132	0.822	0.000	0.828	0.033	0.184	0.358	0.425	0.736	0.449	0.330	0.005	—
Kenya	2022	42253	204	0.660	0.300	0.374	0.229	0.274	0.288	0.210	0.601	0.533	0.480	< 0.001	0.022
Lesotho	2014	9540	197	0.694	0.385	0.325	0.319	0.260	0.236	0.185	0.500	0.573	0.720	0.002	0.074
Liberia	2020	12143	27	0.657	0.331	0.399	0.279	0.224	0.257	0.240	0.485	0.653	0.620	< 0.001	0.003
Madagascar	2021	27788	61	0.677	0.273	0.262	0.305	0.249	0.235	0.212	0.484	0.576	0.600	< 0.001	0.004
Malawi	2016	31945	231	0.767	0.256	0.216	0.311	0.269	0.264	0.156	0.498	0.610	0.610	0.001	0.027
Maldives	2017	11356	432	0.650	0.233	0.138	0.187	0.276	0.317	0.219	0.740	0.336	0.400	0.006	0.097
Mali	2018	14800	273	0.698	0.213	0.332	0.269	0.255	0.276	0.200	0.417	0.668	0.440	0.001	0.013
Mauritania	2020	20237	293	0.738	0.300	0.525	0.282	0.249	0.255	0.214	0.540	0.605	0.340	0.003	0.069
Mozambique	2023	18358	159	0.711	0.277	0.434	0.319	0.269	0.231	0.181	0.461	0.477	0.430	0.001	0.044
Myanmar	2016	17469	847	0.732	0.327	0.290	0.205	0.233	0.298	0.264	0.567	0.512	0.280	0.006	0.113
Nepal	2022	19735	181	0.751	0.242	0.543	0.265	0.256	0.269	0.210	0.601	0.495	0.570	0.001	0.037
Nigeria	2018	54578	346	0.762	0.280	0.408	0.263	0.242	0.273	0.222	0.530	0.675	0.500	0.001	0.019
Pakistan	2018	18580	70	0.804	0.000	0.488	0.083	0.293	0.380	0.244	0.535	0.557	0.430	0.005	—
Papua New Guinea	2017	21911	1189	0.667	0.309	0.263	0.257	0.267	0.285	0.191	0.553	0.821	0.640	0.009	0.156
Philippines	2022	27785	793	1.000	0.409	0.407	0.281	0.228	0.253	0.238	0.710	0.388	0.550	0.001	0.073
Rwanda	2020	21093	58	0.691	0.424	0.239	0.297	0.222	0.272	0.209	0.535	0.400	0.220	< 0.001	0.005
Senegal	2023	21382	147	0.739	0.321	0.46	0.308	0.243	0.252	0.197	0.517	0.505	0.680	0.002	0.022
Sierra Leone	2019	22653	76	0.686	0.343	0.406	0.288	0.243	0.255	0.213	0.457	0.615	0.650	0.001	0.010
South Africa	2016	12109	375	0.702	0.607	0.562	0.249	0.254	0.256	0.241	0.711	0.420	0.790	0.008	0.057
Tajikstan	2017	10643	198	1.000	0.230	0.392	0.249	0.284	0.263	0.204	0.659	0.283	0.110	< 0.001	0.087
Tanzania	2022	20687	389	0.728	0.315	0.349	0.283	0.264	0.257	0.196	0.532	0.513	0.340	0.003	0.048
Togo	2014	13860	45	0.681	0.308	0.371	0.257	0.259	0.268	0.217	0.502	0.593	0.470	< 0.001	0.008
Turkey	2019	7313	121	1.000	0.251	0.714	0.188	0.224	0.312	0.276	0.838	0.286	0.320	0.002	0.053
Uganda	2016	23719	82	0.776	0.281	0.232	0.312	0.275	0.247	0.166	0.531	0.535	0.360	< 0.001	0.015
Zambia	2019	25630	153	0.530	0.363	0.389	0.306	0.251	0.239	0.205	0.571	0.529	0.550	< 0.001	0.019
Zimbabwe	2015	18325	65	0.543	0.342	0.435	0.307	0.248	0.264	0.181	0.544	0.532	0.280	< 0.001	0.018

Notes: Year = Year of data collection, HDI = Human Development Index, GII = Gender Inequality Index, GFS = Global Freedom Score, Married Women = Ever-married women ages 17-49, Single Women = Never-married women ages 15-29
