# Supplementary material for: Prevalence and predictors of childfree people in developing countries
Source: PLoS One. 2025 Nov 12;20(11):e0333906. doi: 10.1371/journal.pone.0333906 (PMC12611150; doi:10.1371/journal.pone.0333906)
Supplement: Attachment — Submitted filename: Response letter.pdf [file pone.0333906.s002.pdf]

Summary of Revisions to "Prevalence and predictors of childfree people in developing countries" (PONE-D-25-19934)

Dear Dr. Ortega and anonymous peer reviewers,

Thank you for your helpful comments on our submission titled "Prevalence and predictors of childfree people in developing countries," and for the opportunity to submit a revision. We describe below how we have addressed each of your concerns, and believe these revisions have substantially strengthened the manuscript.

1 Reviewer #1

"Given the global decline in fertility rates, this study addresses an important and under-researched topic. I have several suggestions for the authors to consider."
We are happy to hear that you found the study important, and believe it addresses a significant topic. We appreciate your very helpful suggestions, and describe how we incorporated them below.

1.1 One of the motivations for examining factors associated with childfree individuals in developed countries is to better understand declining fertility rates and inform pro-natalist policy responses. Could the authors provide more context on fertility trends in the developing countries included in this study? Are these countries also experiencing declining fertility?

We appreciate the opportunity to clarify the study's motivations; Reviewer #3 also asked for this (see 3.4). In this revision, we have completely re-written the background section, and in the new 'Childfree populations in developing countries' subsection, we situate the study as responding to empirical questions raised by the past empirical work on this population.

We briefly mention declining fertility as one reason researchers and media are increasingly interested in childfree people, and as a focus of Second Demographic Transition theory. However, we believe this new section will clarify that declining fertility rates are not a primary motivation for this study.

1.2 I recommend that the authors include the original survey questions used to identify childfree individuals in this study.

We have expanded our discussion of the two variables used to identify childfree respondents to provide additional information:

> We use two variables available in the DHS to identify childfree people. First, `v201` (for women) or `mv201` (for men) measures the total number of children born to a respondent. The value of this variable is not obtained via a single survey question, but instead is computed based on a lengthy series of questions about the respondent's reproductive history. Second, `v613` (for women) or `mv613` (for men) captures respondents' numeric response to the question (in the local language): "*What is the ideal number of children you would like (or 'would have liked', if infecund) to have in your whole life, irrespective of the number of children*

you already have?"

1.3 It is unclear why gender interaction analyses were conducted only for country-level factors, and not for individual-level factors. Given the likelihood that individual-level predictors may have differential effects by gender, the authors should consider testing gender interactions for these variables as well.

The inclusion of cross-level gender interactions had a limited theoretical basis. Additionally, as Reviewer #2 (see 2.4) and Reviewer #3 (see 3.14) noted, these interactions had negligibly small effects with limited practical significance. For these reasons, we have removed gender interactions from the model in this revision.

1.4 Age is currently dichotomised as under 30 versus 30 or older. If the data permit, a more detailed categorisation (e.g. 18–29, 30–39, and 40–49) would allow for a more nuanced interpretation of age-related patterns.

Thank you for this helpful suggestion; Reviewer #3 suggested something similar also (see 3.10.6). In this revision, we now model age in quartiles: under age 22, age 22-29, age 30-39, and over age 39.

1.5 The authors should reconsider how country-level variables are modelled and interpreted. Including them as continuous variables ranging from 0 to 1 means that the reported odds ratios represent comparisons between theoretical extremes (e.g. 0 vs. 1), which may be unrealistic. Rescaling these variables to a 0–100 scale or treating them as categorical may enhance interpretability.

We have retained our 0-1 scaling of the country-level variables for two reasons. First, this is the scaling of HDI and GII used by the United Nations in their computation and reporting of these characteristics, and thus we believe it will be more familiar to readers. Second, this scaling ensures that all the variables in the model have a similar scale, which is essential for the numerical stability of the estimates.

To ensure that the estimates are correctly interpreted, Figure 2 illustrates the predicted probability that a single woman age 22-29 is childfree, over the full range of country-level predictors. In each panel of this figure, we highlight the countries with the minimum, median, and maximum value of each country-level predictor. Additionally, this plot *does not* display predicted probabilities outside the ranges observed in this sample (e.g., at the theoretical extremes), which we agree would be unrealistic.

1.6 The discussion would benefit from a comparison of the findings with those from similar studies conducted in developed countries.

Thanks for this helpful suggestion. There are relatively few such studies, however Reviewer 3 pointed us to a couple (see 3.1). We have now expanded our discussion of the relationship between this study and past work.

1.7 In the results, the authors mentioned 'due to the design and sampling strategy used by the DHS, the majority of these individuals were currently or formerly married.' It would be helpful to provide more detail on the DHS sampling strategy and discuss how this may affect the generalisability of the findings.

Reviewer 3 also asked about this (see 3.10.4). We have clarified the issue by adding the following discussion:

> Because the DHS is designed primarily as a survey to collect fertility data, some countries' samples are restricted to only women, or to only currently or formerly married people. As a result, a majority of the sample were currently or formerly married (72.75%) women (78.15%). However, the over-representation of married women in the total sample does not impact our prevalence estimates, which are restricted to specific population subgroups, and does not impact our analysis of country-level characteristics, which includes gender and marital status as covariates.

1.8 I was unable to locate the supplementary materials referenced in the manuscript.

We apologize for the difficulty in locating the supplementary materials. The supplementary materials, including supplementary information and replication code, are located in the Open Science Foundation repository at `https://osf.io/9zjax/`. We provide this link in the manuscript itself, and have also supplied it in the PLOS One submission system.

2 Reviewer #2

"It was a pleasure to read and provides an important perspective on an emerging topic in LMIC. Great job on this very interesting paper."
We appreciate your positive reaction to the paper, and found your comments very helpful for clarifying the main points. We describe how we incorporated your comments below.

2.1 Line 46: The last sentence of this paragraph doesn't clarify the purpose of the focus on countries' human development, gender inequality, and political freedoms. We can posit that the purpose is to focus on these aspects in relation to the levels of childfree individuals, but this needs to be clarified.

We appreciate the opportunity to clarify the rationale for focusing on development, equality, and freedom; Reviewer #3 also asked for this (see 3.4). In this revision, we have completely re-written the background section, and in the new 'Childfree populations in developing countries' subsection, justify this focus with reference to past empirical research (Verkroost and Monden, 2022) and Second Demographic Transition theory (Lesthaeghe, 2014).

2.2 Definition of childfree: If you are using the definition of childfree as described, there are a few things that need to be discussed either in the methods section or in the limitations. Both number of children born and number of ideal children are time varying and could be influencing each other in either direction. For example, if a woman (or man) is infertile and is not able to have children, that inability to have children may influence the ideal number of children that a person would report. The persons report may be biased to reflect the status quo. I don't think this needs to change your definition, but is worth mentioning in the limitations.

We appreciate your encouragement to clarify our definition of 'childfree' (also see 3.9). In the new 'definitions' section, we now offer a more detailed definition, and discuss its key features.

Reviewer #1 helpfully asked that we clarify the wording of the questions used to identify childfree respondents (see 1.2). To determine a respondent's ideal number of children, they were asked "What is the ideal number of children you would like (or 'would have liked', if infecund) to have in your whole life, irrespective of the number of children you already have?" We use this question in part because, unlike other questions respondents were asked about wanting children, it explicitly asks infecund respondents about the number of children they would have liked to have.

2.3 Methods section: I would appreciate some clarification in this section. I believe that the within-country estimates were calculated by country, but the multilevel regression was estimated on a dataset with all the countries combined, but this is not described anywhere. I would want to know how the individuals in that pooled dataset were weighted as well.

We have replaced the 'estimation' subsection with the 'analysis plan' subsection, where we now provide detailed information about how we achieve each research aim.

2.4 Interactions: The cross-level interactions are included in Table 2, but is not discussed anywhere in the paper, in the results or discussion. It would be useful to present these along with their interpretation.

The inclusion of cross-level gender interactions had a limited theoretical basis, and as Reviewer #3 noted (see 3.14, these interactions had negligibly small effects with limited practical significance. For these reasons, we have removed gender interactions from the model in this revision.

3 Reviewer #3

"This is potentially valuable work, especially because childlessness, regardless of being voluntarily or not, has important implications for fertility decline, population aging, and policy responses."
We are happy to hear that you believe this work may have value and addresses an important topic. We appreciate your detailed comments and suggestions, and have aimed to incorporate each of them in this revision.

3.1 The authors claim that research on childfree people in developing countries has been "largely neglected." This is a bold claim that should be softened. Voluntary childlessness has been studied before in developing contexts, though several key studies are missing from the literature review. Such as de Talancé (2019); Poston et al. (1983); Bagi (2023); Ibisomi and Mudege (2014).

We have restated this claim as: "*Despite decades of research in developing countries on fertility (Tesfa et al., 2023; Bongaarts, 2008; Adsera, 2004; Bongaarts, 2002; Mauldin et al., 1978), which combines voluntary and involuntary childlessness into a single category and focuses broadly on whether or when individuals have children, quantitative demographic research on childfree people remains limited.*" This more nuanced claim is restricted in scope to noting the limited quantitative demographic research on childfree people, while also recognizing the broader literature on fertility more generally.

In addition to clarifying this claim, we have returned to the literature to identify any prior work that we may have missed, and appreciate you pointing us to these references. We have chosen not to incorporate de Talancé (2019) and Bagi (2023) because they combined voluntary and involuntary childlessness into a single category. We also considered incorporating Perez (2021) because they discuss voluntary childlessness in Colombia, however on closer inspection they do not distinguish voluntary and involuntary childlessness in their analyses. Our updated review of prior quantitative research on childfree people in developing countries now includes Poston and Trent (1982), Poston et al. (1983), Ibisomi and Mudege (2014), Razeghi-Nasrabad et al. (2020) Verkroost and Monden (2022), and Chua et al. (2025).

3.2 In light of point 1, the real novelty of this paper is rather in the cross-national DHS-based estimates and predictors, and the authors should say so clearly.

We appreciate the encouragement to more clearly state the aims of this study. In the new 'Research Aims' section, we now explicitly state the study's three aims:

> Guided by this past research Poston et al. (1983); Razeghi-Nasrabad et al. (2020); Chua et al. (2025); Verkroost and Monden (2022) and theory Lesthaeghe (2014); Zaidi and Morgan (2017); Poston and Trent (1982), the current study has three research aims. First, to facilitate this and future childfree research, we pursue the methodological aim of developing software designed to import, recode, and harmonize demographic data on childfree people (**Aim #1**). Second, we pursue the descriptive aim of estimating and comparing the country-level prevalence of childfree people in a large sample of developing countries (**Aim #2**). Third, we pursue the exploratory aim of identifying country-level characteristics that are associated with a person being childfree (**Aim #3**).

We now use these aims to organize presentation of methods and results also.

3.3 The biggest weakness is that the paper does not clearly explain why these findings matter. What does a 1.9% prevalence mean for population trends? Does this indicate early signs of the Second Demographic Transition spreading to the Global South? Or is voluntary childlessness still too rare to have demographic consequences? What are the main predictors? What does it mean for policy or future implication of demographic trends? A strong revision should answer these questions directly to allow a clear take-home message of the study.

We have completely re-written the discussion section, which we now organized around each of our three research aims, in each case highlighting the significance of our findings:

- Aim #1 - "*It is significant because it simplifies not only research on childfree people in the developing world, but also cross-national and longitudinal research on this population. Since this function was added to the package in June 2024, it has been downloaded and installed nearly 3000 times.*"
- Aim #2 - "*These findings are significant for two reasons. First, these are the first cross-nationally comparable estimates of childfree prevalence in the 21st century, and provide a benchmark against which changes in prevalence can be evaluated. Second, they highlight that some developing countries have large populations of childfree people, and that childfree people are as common in some developing countries as they are in developed countries. Thus, they suggest that being childfree is not a choice restricted to those living in the West or in wealthy countries.*"
- Aim #3 - "These findings are significant because they extend support for the role of human development on reproduction, from its previously documented effects on fertility (i.e., women have fewer children on average), to its effects on being childfree (i.e., more people do not want to have any children)."

Because these data are cross-sectional, following recommendations from Zaidi and Morgan (2017) in their critique of Second Demographic Transition theory, we refrain from speculating about what these findings may mean for demographic transitions. Because there is substantial cross-national variation not only in the prevalences we estimate, but also in the local cultural and political contexts, we refrain from offering policy implications.

3.4 I was surprised by the lack of theoretical grounding. The introduction frames the study around the "unique needs" of childfree people, rather than focusing on individual- or population-level implications of childlessness.

We appreciate the opportunity to clarify the study's theoretical grounding; Reviewer #1 also requested greater clarity on the study's motivations (see 1.1). In this revision, we have completely re-written the background section, and in the new 'Childfree populations in developing countries' subsection, we frame the study as responding to empirical questions raised by the past empirical work (e.g., Verkroost and Monden, 2022), which we do with reference to Second Demographic Transition theory (Lesthaeghe, 2014).

3.5 The fact that there is no explicit theoretical framework also tone down the choice of macro-level predictors, which seems rather arbitrary. The omission of Second Demographic Transition (SDT) theory is particularly notable. SDT offers a clear rationale for expecting links between development, gender equality, political freedoms, and voluntary childlessness, as it emphasizes individualization, gender equity, and post-materialist values. The lack of theoretical grounding makes the selection of HDI, GII, and GFS appear ad hoc.

We agree that the rationale for focusing on these three country-level variables was unclear. In the new 'Childfree populations in developing countries,' we use Second Demographic Transition theory to inform our focus on development, equality, and freedom. In the revised 'Methods' section, we more clearly identify HDI, GII, and GFS as operationalizations of these SDT-hypothesized constructs.

3.6 The authors repeatedly define childfree individuals as those who "do not have or want children." The standard demographic definition is "do not have AND do not want children." This should be corrected everywhere.

The phrase "do not have or want children" has been used for decades to define childfree individuals (e.g., Jacobson and Heaton, 1991; Lemke et al., 2023; Hewitson, 2007), and occurred in our original manuscript in two parenthetical references. However, to avoid any ambiguity, we have removed these two instances.

3.7 The choice of DHS data raises significant concerns. While DHS is valuable for fertility research, it is not ideally suited to studying voluntary childlessness. The paper does not discuss this limitation adequately. DHS samples differ across countries—some include only married women—making cross-national comparability problematic. The authors acknowledge this briefly but proceed to make sweeping cross-country statements.

We believe DHS data is ideally suited to study childfree people because it is one of the few representative demographic surveys that contains questions necessary to identify childfree people (e.g., an "ideal number of children" question).

You are correct that the variation in sample universes across countries raises some challenges to comparing countries using these data. We now explicitly address this issue, and describe how we solve it, in the newly-added 'analysis plan' section:

> Because the universe of DHS samples varies across countries (e.g., only married women were surveyed in some countries), it is not possible to compute cross-nationally comparable prevalence estimates for complete country populations. Instead, we compute cross-nationally comparable prevalence estimates for two subgroups included in all or most countries' sample universes. First, we estimate the percentage of ever-married women ages 17–49 who are childfree. This is the largest population subgroup for which a cross-nationally comparable estimate can be computed for all 51 countries. Second, we estimate the percentage of single women ages 15–29 who are childfree. Members of this population subgroup engage in particularly significant reproductive decision making, and

cross-nationally comparable estimates can be computed for all but 5 countries (Afghanistan, Bangladesh, Egypt, Jordan, and Pakistan).

3.8 Given point 7, previous studies who make use of DHS-based estimates for childlessness in developing countries (such as Chua et al. (2025)) or other studies that use different data should be used as benchmark.

In the new 'Childfree populations in developing countries' section, we explain that although country-level prevalence estimates are available for 16 developing countries, they cannot be used as comparative benchmarks because they were computed at different times and using different operationalizations, which limits their comparability. Obtaining cross-nationally comparable prevalence estimates is a key motivation and goal of this study.

3.9 The operationalization of childfree status is also problematic. Respondents reporting 0 ideal children are classified as childfree, but ideal fertility preferences, particularly among young people, often change under social pressure. The authors briefly acknowledge that DHS is cross-sectional but do not explain why this is a critical limitation for interpreting childfree as a life-course outcome.

We appreciate your encouragement to clarify our definition of 'childfree' (also see 2.2). In the new 'definitions' section, we now offer a more detailed definition, and discuss its key features.

3.10 Overall, the methodological details given to the reader are insufficient to ensure reproducibility. The supplementary information reference in the text is unavailable for review, and no detailed country-level table with sample size, survey year, percentage of childlessness in country-year is given.

We apologize for the difficulty in locating the supplementary materials. The supplementary materials, including supplementary information and replication code, are located in the Open Science Foundation repository at `https://osf.io/9zjax/`. We provide this link in the manuscript itself, and have also supplied it in the PLOS One submission system. The requested country-level table is provided in the supplementary information provided in that repository.

3.10.1 The time frame of the data is ambiguous. The authors state they used the "most recent DHS from the past 10 years," but it is unclear whether data were pooled across multiple rounds or restricted to one survey per country. This ambiguity undermines claims about temporal trends, such as an "emerging childfree population," which cannot be inferred from cross-sectional data.

We now clarify that: "When multiple waves of these data were available from a single country, we use only the most recent wave." Thus, our complete dataset is cross-sectional and contains one wave of data for each country. The *Supplementary Information*, which is available at `https://osf.io/9zjax/`, provides detailed information about each country, including the year of data we used in each case.

3.10.2 35,854 respondents with "undetermined" status were excluded without explanation. Were they disproportionately young, unmarried, or missing key variables?

We now explain that: "*An additional 35,854 people were excluded because they were missing one or both of the variables used to determine whether they were childfree, and therefore their childfree status could not be determined.*" These excluded individuals represent less than 2% of the analytic sample, and their exclusion does not impact the sample's demographic composition.

3.10.3 As far as I understand, infecund individuals are recoded in data, but not clearly excluded from the analyses. This conflates voluntary and involuntary childlessness.

The DSH data do contain information about infecundity. However, because we use the definition of childfree defined by the ABC framework (Neal and Neal, 2024), this information is not relevant for determining whether an individual is childfree. Specifically, we define childfree individuals as those who do not have children and do not want to have children, regardless of their fecundity. An infecund individual who does not want children, even if having them were possible, is childfree.

3.10.4 The sample is strongly unbalanced with 78% of respondents being women, yet no justification is given.

Reviewer 1 also asked about this (see 1.7). We have clarified the issue by adding the following discussion:

> Because the DHS is designed primarily as a survey to collect fertility data, some countries' samples are restricted to only women, or to only currently or formerly married people. As a result, a majority of the sample were currently or formerly married (72.75%) women (78.15%). However, the over-representation of married women in the total sample does not impact our prevalence estimates, which are restricted to specific population subgroups, and does not impact our analysis of country-level characteristics, which includes gender and marital status as covariates.

3.10.5 The model omits key individual-level predictors such as education, wealth, and urban/rural residence, all known to correlate with voluntary childlessness.

The DHS data includes respondents' educational attainment by level (e.g., primary, secondary) and by year. However, because educational levels and years vary across countries, these variables are not suitable for cross-national comparisons and have not been harmonized by DHS for this purpose.

The DHS data include a wealth index that is constructed using principal components, and is difficult to directly interpret. Additionally, it has been critiqued for being invalid in samples with both urban and rural populations. The most recent wave contains an attempted correction for urban/rural wealth disparities, but it has not been validated and is not available in earlier waves.

Although it is not possible to control for education or wealth, we now include respondents'

urbanicity as a predictor. It has a statistically significant association with being childfree, but like some of the other predictors (see 3.14), the effect size is very small.

3.10.6 Age is modeled very crudely as a binary variable (less than 30 vs 30 or greater), which ignores well-known life-course patterns in fertility decision making. Demographic research typically models age continuously or in 5-year reproductive cohorts to distinguish temporary postponement among younger people from permanent childfree status at older ages. The authors should consider using finer age groups or, at minimum, present age-specific prevalence for older cohorts (e.g., 40-49) to better reflect permanent voluntary childlessness.

Thank you for this helpful suggestion; Reviewer #1 suggested something similar also (see 1.4). In this revision, we now model age in quartiles: under age 22, age 22-29, age 30-39, and over age 39.

3.10.7 HDI, GII, and GFS are highly correlated, yet no multicollinearity diagnostics (e.g., VIFs) are presented.

Correlation among independent variables does not affect the properties of the estimators; they remain BLUE (best linear unbiased estimates). Correlation among independent variables can reduce statistical power, making it more difficult to detect statistically significant effects. However, as you correctly note in 3.14, our sample is very large, and consequently all of our estimates are already statistically significant. For these reasons, we follow the recommendation of leading econometricians Goldberger (1991) and Wooldridge (2014) to ignore multicollinearity.

3.10.8 Other relevant indicators, such as maternal mortality, are discussed but ignored in the models. Are they available? If yes, why are they excluded? (this also recalls the need of a theory or a research questions guiding the analyses)

There are two primary limitations to including other potential country-level variables. First, many of the other variables we considered (e.g., maternal mortality, HIV, climate risk) were not available for all the countries in our sample, or were not available for the years of our sample. Second, fitting a GLMM with over 2 million observations is computationally intensive with even a small number of country-level variables, and adding additional variables leads to impractically long estimation times or a lack of model convergence.

In this revision, we have re-written the background section to more clearly state our research aims, describing our model as exploratory and motivating our choice of country-level variables with reference to prior research and theory. In addition, in the discussion section, we comment on other country-level characteristics that should be explored in future research.

3.11 The reported childfree prevalence cannot be generalized to entire populations because the DHS sample is heavily skewed toward women and married individuals. The authors should emphasize this limitation. For instance, Indonesia's DHS-based childfree prevalence (0.4%) is higher than its reported permanent childlessness (0.22%) in the Indonesian Family Life Survey (de Talancé, 2019). By definition, voluntary childlessness should not exceed total childlessness. The authors should conduct plausibility checks against external sources and discuss possible reasons for these discrepancies.

You are certainly correct that the DHS sampling strategy means that samples disproportionately include certain individuals (e.g., women, married individuals). In fact, data in some countries were collected *only* from women, or *only* from married individuals. To clarify the issue, in the newly-added 'Analysis Plan' section, we now explain:

> Because the universe of DHS samples varies across countries (e.g., only married women were surveyed in some countries), it is not possible to compute cross-nationally comparable prevalence estimates for complete country populations. Instead, we compute cross-nationally comparable prevalence estimates for two subgroups included in all or most countries' sample universes. First, we estimate the percentage of ever-married women ages 17–49 who are childfree. This is the largest population subgroup for which a cross-nationally comparable estimate can be computed for all 51 countries. Second, we estimate the percentage of single women ages 15–29 who are childfree. Members of this population subgroup engage in particularly significant reproductive decision making, and cross-nationally comparable estimates can be computed for all but 5 countries (Afghanistan, Bangladesh, Egypt, Jordan, and Pakistan).

There are many reasons that the estimates we report in this study might differ from estimates reported in other studies, including differing definitions, differing sample universes, and differing times. In the case of Indonesia, our prevalence estimate of 0.4% refers to *current* childfree status among single women ages 15–29 in *2017*. In contrast, the 0.22% estimate reported by de Talancé (2019) refers to *permanent* childfree status for women *with a range of ages and partnership statuses* in *2014*. These two estimates were computed using such different methods and data that it is difficult to directly compare them or speculate about why they might differ.

3.12 The authors report that almost half of the total sample is under age 30 and that childfree prevalence is highest among younger women (15-29 years). Figure 1 explicitly focuses on single young women, where prevalence reaches over 5% in some countries. This suggests that childfree status is disproportionately concentrated among younger individuals in the DHS sample. However, the multilevel model reports: "Those under age 30 (B = -0.271, OR = 0.762, p<0.001) were less likely to be childfree." This means that, after controlling for other factors, younger people are less likely to be childfree than older people. This discrepancy likely reflects conceptual and methodological issues: (1) the classification of young respondents with 0 ideal children as childfree, even though many may later have children; (2) strong confounding by marital status, which reverses the age effect once controlled for; and (3) the skewed sample composition. The authors should clarify this contradiction, ideally by providing age-specific prevalence and discussing the life-course dimension of voluntary childlessness.

Figure 1 reports the prevalence of being childfree among single women age 15–29 for the sake of comparing national prevalence rates in a cross-nationally comparable subpopulation. It is not possible to conclude from the values in this figure (or the associated table in the *Supplementary Information*) that childfree status is concentrated among younger individuals.

Table 2 reports the estimates from a generalized linear mixed model, and these estimates can be used to draw conclusions about demographic subgroups where childfree status may be more common. These results *do not* suggest that childfree status is more common among young people generally. Instead, they indicate that childfree status is most common among older people (over age 39), and least common among people in prime reproductive years (age 22-29). These results also indicate that childfree status is not substantially less common among women than men, but it is much more common among single people than married people. Collectively, these results suggest that the prevalence values shown in Figure 1 are higher than for the general population not because they are computed with reference to a younger or female population, but because they are computed with respect to a never-married population.

3.13 The explanation offered for the fact that older people are more likely to be childfree - that younger people "receive messages about the importance of having children" - is vague and unconvincing. A life-course explanation (temporary postponement vs permanent childlessness) would be more appropriate.

In this revision, we have clarified that:

> The individual-level characteristics are not of direct interest for research aim #3, but were included to isolate the independent association of childfree status with the country-level characteristics.

Therefore, we no longer discuss or interpret them, except to note (following your suggestion in 3.14) that:

> The strength of the associations between childfree status and these individual-level demographic characteristics was generally small. Marital status is an ex-

ception and had a strong association with childfree status, which is consistent with prior studies in the United States (Neal and Neal , 2023), Japan (Neal and Neal, 2024), and the Philippines (Chua et al., 2025).

3.14 Many of the reported associations, particularly for HDI, GII, and GFS, are statistically significant but substantively trivial given the very large sample size. For example, gender differences in childfree prevalence at high HDI levels (Figure 2A) and slopes for gender inequality (Figure 2B) and political freedom (Figure 2C) are extremely small and should be interpreted cautiously. Moreover, confidence intervals are missing from the figures, making it impossible to judge the reliability of these estimates. The authors should include uncertainty intervals, present predicted probabilities, and tone down claims of meaningful macro-level effects.

We completely agree that the statistical significance of our estimates is trivial given the large sample size, and that interpretation should instead focus on effect sizes. In this revision, we have (1) revised our interpretation of the estimates to recognize their small effect sizes and (2) eliminated gender interactions which had a limited theoretical basis and negligibly small effects.

We have revised Figure 2 to display 99% confidence intervals.

3.15 The paper fails to emphasize that DHS only captures current fertility preferences, not stable life-course decisions. Statements about an "emerging" childfree population are unjustified.

We completely agree that these data only capture respondents' *current* family status. We have aimed to clarify this intention in the new 'definitions' section, where we offer a more detailed definition of childfree. In addition, we have taken care to eliminate any language (e.g., "emerging") that implies a temporal or causal dimension to this work.

3.16 Be aware that there are multiple typographical errors (e.g., "its' members") and informal phrases ("want questions") that should be corrected before a second submission.

We have carefully proofread this revision.

4 Editor

4.1 Please ensure that your manuscript meets PLOS ONE's style requirements.

We have prepared our manuscript using the PLOS One LaTeX template, and have aimed to follow all the style requirements in this revision.

4.2 Please note that funding information should not appear in the Acknowledgments section or other areas of your manuscript.

We have removed information about funding from the Acknowledgements section. Our current Funding Statement is correct.

4.3 We note that Figure 1 in your submission contain [map/satellite] images which may be copyrighted.

The coordinated used to draw country borders was obtained from the public domain Natural Earth project. The terms of use located at the above link specify: "No permission is needed to use Natural Earth. Crediting the authors is unnecessary."

4.4 If the reviewer comments include a recommendation to cite specific previously published works, please review and evaluate these publications to determine whether they are relevant and should be cited. There is no requirement to cite these works unless the editor has indicated otherwise.

In our responses above, we discuss each reference suggested by reviewers and either note where we have incorporated it, or explain why we have chosen not to incorporate it.

References

Adsera, A. (2004). Changing fertility rates in developed countries. the impact of labor market institutions. *Journal of population economics*, 17(1):17–43.

Bagi, M. (2023). Prevalence, reasons and consequences of childlessness in the world and iran: a systematic review. *Journal of Population Association of Iran*, 18(35):97–148.

Bongaarts, J. (2002). The end of the fertility transition in the developed world. *Population and development review*, pages 419–443.

Bongaarts, J. (2008). Fertility transitions in developing countries: Progress or stagnation? *Studies in family planning*, 39(2):105–110.

Chua, A. L. B., Neal, J. W., and Neal, Z. P. (2025). Trends in the prevalence of childfree women in the Philippines, 1993–2022. *Journal of Family Issues*.

de Talancé, M. (2019). Education, fertility and childlessness in indonesia. *Erudite Working Papers*, 15-2019.

Goldberger, A. S. (1991). *A course in econometrics*. Harvard University Press.

Hewitson, G. (2007). Feminist economics as a postmodern moment. *Review of Social Economy*, 65(2):187–193.

Ibisomi, L. and Mudege, N. N. (2014). Childlessness in nigeria: perceptions and acceptability. *Culture, Health & Sexuality*, 16(1):61–75.

Jacobson, C. K. and Heaton, T. B. (1991). Voluntary childlessness among american men and women in the late 1980's. *Social biology*, 38(1-2):79–93.

Lemke, J., Mollen, D., and Buzolits, J. S. (2023). Sterilized and satisfied: Outcomes of childfree sterilization obtainment and denials. *Psychology of Women Quarterly*, 47(4):562–573.

Lesthaeghe, R. (2014). The second demographic transition: A concise overview of its development. *Proceedings of the National Academy of Sciences*, 111(51):18112–18115.

Mauldin, W. P., Berelson, B., and Sykes, Z. (1978). Conditions of fertility decline in developing countries, 1965-75. *Studies in family planning*, 9(5):89–147.

Neal, J. W. and Neal , Z. P. (2023). Prevalence, age of decision, and interpersonal warmth judgements of childfree adults: Replication and extensions. *PLOS One*, 18(4):e0283301.

Neal, Z. P. and Neal, J. W. (2024). Sample, time, and wording effects on estimating the prevalence of childfree adults: Insights from Japan. *PLOS One*, 19(4):e0302184.

Neal , Z. P. and Neal, J. W. (2024). A framework for studying adults who neither have nor want children. *The Family Journal*, 32(1):121–130.

Perez, C. M. (2021). Male childlessness across the adult life course: A case study addressing a potentially 'vulnerable' population. *Advances in Life Course Research*, 49:100414.

Poston, D. L., Kramer, K. B., Trent, K., and Yu, M.-Y. (1983). Estimating voluntary and involuntary childlessness in the developing countries. *Journal of Biosocial Science*, 15(4):441–452.

Poston, D. L. and Trent, K. (1982). International variability in childlessness: A descriptive and analytical study. *Journal of Family Issues*, 3(4):473–491.

Razeghi-Nasrabad, H. B., Abbasi-Shavazi, M. J., and Moeinifar, M. (2020). Are we facing a dramatic increase in voluntary and involuntary childlessness in iran that leads to lower fertility? *Crescent Journal of Medical & Biological Sciences*, 7(2).

Tesfa, D., Tiruneh, S. A., Gebremariam, A. D., Azanaw, M. M., Engidaw, M. T., Kefale, B., Abebe, B., Dessalegn, T., and Tiruneh, M. (2023). The pooled estimate of the total fertility rate in Sub-Saharan Africa using recent (2010–2018) Demographic and Health Survey data. *Frontiers in Public Health*, 10:1053302.

Verkroost, F. C. and Monden, C. W. (2022). Childlessness and development in Sub-Saharan Africa: Is there evidence for a U-shaped pattern? *European Journal of Population*, 38(3):319–352.

Wooldridge, J. M. (2014). *Introduction to econometrics*. Cengage Learning.

Zaidi, B. and Morgan, S. P. (2017). The second demographic transition theory: A review and appraisal. *Annual review of sociology*, 43(1):473–492.
